# Genetic Variants in the *FGB* and *FGG* Genes Mapping in the Beta and Gamma Nodules of the Fibrinogen Molecule in Congenital Quantitative Fibrinogen Disorders Associated with a Thrombotic Phenotype

**DOI:** 10.3390/ijms21134616

**Published:** 2020-06-29

**Authors:** Tomas Simurda, Monika Brunclikova, Rosanna Asselta, Sonia Caccia, Jana Zolkova, Zuzana Kolkova, Dusan Loderer, Ingrid Skornova, Jan Hudecek, Zora Lasabova, Jan Stasko, Peter Kubisz

**Affiliations:** 1 National Center of Hemostasis and Thrombosis, Department of Hematology and Transfusiology, Comenius University in Bratislava, Jessenius Faculty of Medicine in Martin and University Hospital in Martin, 03601 Martin, Slovakia; simkovamonika@gmail.com (M.B.); jana.zolkova@gmail.com (J.Z.); inkaskornova@gmail.com (I.S.); hudecek@unm.sk (J.H.); jan.stasko@uniba.sk (J.S.); peter.kubisz@uniba.sk (P.K.); 2 Department of Biomedical Sciences, Humanitas University, 20090 Pieve Emanuele, Italy; rosanna.asselta@hunimed.eu; 3 Humanitas Clinical and Research Center (IRCCS), 20089 Rozzano, Italy; 4 Department of Biomedical and Clinical Sciences”L. Sacco”, Università degli Studi di Milano, 20157 Milan, Italy; sonia.caccia@unimi.it; 5 Biomedical Center Martin, Comenius University in Bratislava, Jessenius Faculty of Medicine in Martin, 03601 Martin, Slovakia; zuzana.snahnicanova@uniba.sk (Z.K.); dusan.loderer@uniba.sk (D.L.); 6 Department of Molecular Biology, Comenius University in Bratislava, Jessenius Faculty of Medicine in Martin, 03601 Martin, Slovakia; zora.lasabova@uniba.sk

**Keywords:** fibrinogen, quantitative fibrinogen disorders, beta and gamma nodules, *FGB* gene, *FGG* gene, mutations associated with thrombosis

## Abstract

Fibrinogen is a hexameric plasmatic glycoprotein composed of pairs of three chains (Aα, Bβ, and γ), which play an essential role in hemostasis. Conversion of fibrinogen to insoluble polymer fibrin gives structural stability, strength, and adhesive surfaces for growing blood clots. Equally important, the exposure of its non-substrate thrombin-binding sites after fibrin clot formation promotes antithrombotic properties. Fibrinogen and fibrin have a major role in multiple biological processes in addition to hemostasis and thrombosis, i.e., fibrinolysis (during which the fibrin clot is broken down), matrix physiology (by interacting with factor XIII, plasminogen, vitronectin, and fibronectin), wound healing, inflammation, infection, cell interaction, angiogenesis, tumour growth, and metastasis. Congenital fibrinogen deficiencies are rare bleeding disorders, characterized by extensive genetic heterogeneity in all the three genes: *FGA*, *FGB*, and *FGG* (enconding the Aα, Bβ, and γ chain, respectively). Depending on the type and site of mutations, congenital defects of fibrinogen can result in variable clinical manifestations, which range from asymptomatic conditions to the life-threatening bleeds or even thromboembolic events. In this manuscript, we will briefly review the main pathogenic mechanisms and risk factors leading to thrombosis, and we will specifically focus on molecular mechanisms associated with mutations in the C-terminal end of the beta and gamma chains, which are often responsible for cases of congenital afibrinogenemia and hypofibrinogenemia associated with thrombotic manifestations.

## 1. Structure and Function of Fibrinogen 

**Figure 1:**
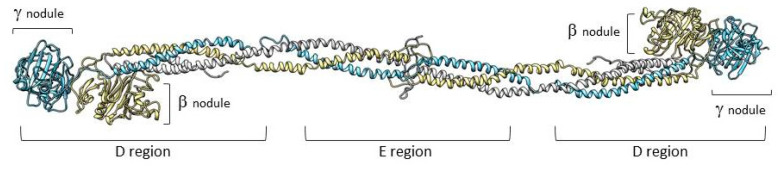
Representation of fibrinogen molecule based on its crystal structure [6]. The three fibrinogen chains (Aα, Bβ, and γ) forming the molecule are shown in white, yellow, and light blue, respectively. The peripheral portions of the D regions are composed of the globular C-terminal nodules of the βB and γ chains.

Fibrinogen is a large complex glycoprotein made up of three pairs of polypeptide chains, designated as Aα (encoded by the *FGA* gene), Bβ (*FGB*), and γ (*FGG*) with a molecular total mass of about 340 kDa. Genes, arranged from the centromere to telomere, are grouped in a cluster that extents ≈ 50 kb on chromosome 4 [1]. The *FGA* gene has a 7.6-kb size and consists of six exons, *FGB* has an 8-kb size, and presents eight exons. Lastly, *FGG* includes an 8.5-kb region and presents 10 exons [2]. The fibrinogen hexameric molecule has a rod-like shape with dimensions of 9 × 47.5 × 6 nm with a negative net charge at physiological pH (isoelectric point: pI = 5.8) [3]. The two end nodules (forming the C-terminal portions of the D regions) are similar and are made of the C-terminal ends of Bβ and γ chains while the center is a slightly smaller nodule (within the E region) that consists of the N-terminal ends of the six polypeptide chains [4,5]. The central nodule is connected to the distal β-nodules and γ-nodules through two elongeted coiled-coil regions (Figure 1).

The Aα, Bβ, and γ chains are kept together in the central nodule by five symmetrical disulfide bridges [7,8]. In this case, we found constitutive binding sites participating in fibrinogen transformation to fibrin, fibrin assembly, establishing of the fibrin network, and platelet functions as well as parts of the molecule that are available after fibrinopeptide release [9]. Fibrinogen biosynthesis, which mainly takes place in hepatocytes, starts with the coordinate transcription of all the three fibrinogen chains. In the endoplasmic reticulum (ER), the three chains are assembled initially into Aαγ and Bβγ dimers, then to AαBβγ trimers, and, lastly, to (AαBβγ)_2_ hexamers. This hexamer is transported to the Golgi complex where post-translation modifications form the mature fibrinogen molecule that is then secreted into the blood [10,11].

The physiological concentration of fibrinogen in plasma is 1.80–4.20 g/L with levels appreciably below or above this range associated with pathological bleeding and/or thrombosis. Fibrinogen has a circulating half-life of ~ 4 days [12,13]. 

Fibrinogen and fibrin are essential in blood clotting, fibrinolysis, cellular and matrix interactions, wound healing, inflammation, angiogenesis, and even in neoplastic processes [14]. These events are regulated by interactions between specific sites on fibrin and fibrinogen and extrinsic molecules such as growth factors, proenzymes, clotting factors, enzyme inhibitors, and cell receptors [15]. During fibrin formation, the N-terminal part of the Aα and Bβ chains (within the E region) are cleaved by thrombin, releasing fibrinopeptides A and B. This cleavage results in the unmasking of four binding sites on the E region in which each can bind to the C-terminal portion of a D region from fibrin monomers [4,16,17]. In this way, monomeric fibrin self-assemblies spontaneously yield fibrin oligomers that lengthen to make two-stranded protofibrils [15]. In addition, fibrin interacts both with platelets to increase the size of the clot as well as with several different proteins and cells, which promotes the inflammatory reaction and the accumulation of cells required for wound repair after injury [18,19]. Specifically, fibrinogen plays a pivotal role in primary hemostasis during which surface interactions between fibrinogen and the glycoprotein (GP) IIb/IIIa receptor of platelets occur [20]. These interactions are facilitated by the release of the intracellular tether of GP IIb/IIIa, possibly via release from cytoskeletal actin components (the so-called inside-out signaling), which allows the extracellular domains of the GP IIb/IIIa receptor complex to expose multiple binding sites for fibrinogen and, also, for the von Willebrand factor [21]. In turn, this event triggers platelet aggregation by bridging GP IIb/IIIa receptors of adjacent activated platelets. Ligand binding and GP IIb/IIIa clustering subsequently activate the outside-in signaling, which initiates and amplifies different cellular platelet processes, i.e., spreading, thrombus consolidation, and clot retraction [21,22].

To summarize, the fibrinogen molecule is implicated in a number of functions as well as complex interactions with other molecules. Therefore, it is not surprising that the association of fibrinogen/fibrin with human disorders can result from an altered triggering of signaling pathways, from alterations in the normal range of fibrinogen levels, or from mutations impacting its structure/function [16,23].

## 2. Congenital Fibrinogen Disorders

### 2.1. Classification

Diseases affecting fibrinogen can be inherited or acquired. Congenital fibrinogen disorders (CFD) are traditionally categorized on the basis of plasma concentration as follows: (i) type 1: quantitative disorders with a reduced level of antigen and functional activity (including afibrinogenemia with a plasma level of fibrinogen lower than 0.1 g/L and hypofibrinogenemia with a plasma level of fibrinogen between 0.1 and 1.8 g/L), or (ii) type 2: qualitative disorders with normal or reduced antigen levels associated with abnormal functional activity (including dysfibrinogenemias and hypodysfibrinogenemias) [24,25,26]. Recommendations of the “Factor XIII and Fibrinogen Subcommittee” and of the “Scientific Standardization Committee” of the “International Society on Thrombosis and Haemostasis” (ISTH) recently proposed both a path to follow for the diagnostic approach to CFD and a new classification of CFD based on the clinical phenotype and on fibrinogen levels. This CFD classification, summarized in Table 1, provides an appropriate tool to clinicians to identify the patients at increased risk of complications, in particular bleeding and/or thrombosis [27].

According to the “Annual Global Survey 2017” of “World Federation Hemophilia” (WFH), which assessed information from 116 countries, fibrinogen deficiencies represent 9.3% of cases of rare bleeding disorders (RBD), which are slighty more prevalent in women when compared to men (1249 vs. 1026, i.e., 54.9% vs. 45.1% in 160 cases where gender was not specified, data from the 68 countries reporting details on CFD) [28]. The estimated prevalence of afibrinogenemia has long been considered to be approximately 1 in 1,000,000 [29,30], though a recent report indicates that the world-wide prevalence for recessively-inherited fibrinogen deficiencies could be up to 10-fold higher than that reported so far [31]. Generally speaking, in populations with frequent consanguineous marriages, the prevalence of afibrinogenemia, and also the occurence of other disorders of hemostasis with autosomal recessive inheritance, is increased. Geographical differences in the prevalence reflect high occurence in children of consanguineous parents in Muslim countries [32].

Concerning specifically Slovakia, the country for which we have acquired a robust experience on RBD and characterized by a population of 5,445,087 individuals, the total number of patients with RBD in 2019 was 1274 (according to the “National Registry of Congenital Bleeding Disorders,” which is run by the National Hemophilia Center, University Hospital, and Medical School of Comenius University in Bratislava). The prevalence for congenital afibrinogenemia was estimated to be 1 in 5,000,000, which means it is extremely rare [30]. Congenital hypofibrinogenemia is generally more frequent than afibrinogenemia, but prevalence is difficult to establish due to the large number of asymptomatic patients. In Slovakia, the prevalence of congenital hypofibrinogenemia was estimated to be 1 in 50,000 [33]. 

### 2.2. Clinical Features

In afibrinogenemia, most patients suffer from major bleedings with a minority that can be asymptomatic. Umbilical cord bleeding in neonates is generally the first and most frequent sign of the disorder manifesting in 85% of the cases [34]. The disorder can manifest by uncommon intracranial bleeding in childhood, which is the principal cause of death in affected patients. Except these potentially life-threatening complications, the most frequent manifestations of afibrinogenemia are mucosal bleeding, especially menorrhagia, epistaxis, and bleeding in the oral cavity [35]. Musculoskeletal bleeding (and also bleeding into the joints) is reported in approximately half of the individuals with afibrinogenemia, and, in some studies, it was more prevalent than bleedings from mucosal surfaces. Bleeding from the gastrointestinal and urinary system occurs less frequently [36,37]. Moreover, quantitative fibrinogen abnormalities can lead to complicated wound healing [10,21]. Besides spontaneous bleeding, bleeding after minor injury and excessive bleeding during various intervenions are further major manifestations of afibrinogenemia [24]. In general, the bleeding phenotype in hypofibrinogenemic patients depends on the fibrinogen plasma value. Above 1 g/L, most patients are completely asymptomatic [38]. 

Paradoxically, patients with afibrinogenemia and hypofibrinogenemia can experience severe, spontaneous, or repeated thromboembolic complications. Arterial and also venous thromboembolic episodes in various locations have been reported, i.e., thrombosis in peripheral arteries, recurrent myocardial infarctions [39,40], thrombosis of abdominal aorta with peripheral embolisation, cerebral [41] or hepatic vein thrombosis [42], or venous thrombosis after delivery [1,43]. Korte and colleagues described in their study 128 patients with CFD and thrombosis. In particular, 25 patients were diagnosed with afibrinogenemia, and 16 were suffering from hypofibrinogenemia. In approximately half of the cases, thromboses were spontaneous, frequently developed at a young age, and were commonly present in large vessels. The recurrence of thrombotic events is not uncommon with trauma, surgery, and parturition significantly contributing to the risk [44]. 

## 3. Pathogenesis and Risk Factors for Thrombosis in Congenital Quantitative Fibrinogen Disorders

The pathogenesis at the basis of the paradoxical thrombotic tendency in congenital quantitative fibrinogen disorders is likely multifactorial, depending on exogenous and endogenous risk factors such as genetic thrombophilia, use of fibrinogen concentrate, trauma, immobilization, or pregnancy. The true mechanisms of thrombosis in these patients still remain unexplained [26,45]. 

In quantitative disorders of fibrinogen, the increased risk of thrombosis can be related to the fact that, from one side, circulating thrombin concentrations are increased in the absence of fibrinogen (lack of the substrate), and from the other to the fact that thrombin is no longer inactivated by fibrin (known in the past as antithrombin I factor) [41]. In the physiological hemostatic process, fibrin clot itself exhibits significant thrombin-binding potential for the concentration of free thrombin in blood plasma decreases due to its binding not only to fibrinopeptide A/B cleavage sites on the fibrinogen molecule but also by binding to fibrinogen through an anion binding site (exosite 1). In addition, antithrombin I also show a significant affinity to the D nodules of fibrin(ogen) molecules containing the γ chain variant termed γ’ [46]. However, in quantitative fibrinogen disorders, some free thrombin remains in the circulation [44] with its level directly depending on the fibrinogen plasma level. Low plasma levels of fibrinogen in hypofibrinogenemic patients can partially suppress thrombin activity. This is considered to be the largest prothrombotic trigger in this group of diseases. However, in addition to free thrombin, prothrombin fragments and high plasma levels of thrombin-antitrombin complexes were also observed in patients with quantitative fibrinogen disorders [26,38,44].

Mutations in the *FGB* gene are of interest since the Bβ chain is considered the rate-limiting factor in the hepatic production of the fibrinogen hexamer [43,47] and, therefore, can result in quantitative fibrinogen disorders due to impaired fibrinogen secretion. In quantitative fibrinogen disorders, mutant chain in the βC domain is retained inside the cell and only hexamers containing the normal chain are secreted [48]. It may be that this subset of mutations can determine the formation of hexamers that partially escape the endoplasmic reticulum quality control degradation pathway and, thus, can be found in the circulation, albeit at low levels. In these cases, even low levels of mutated fibrinogen could contribute to a hypercoagulable state by affecting fibrin clot properties such as the fibrinolysis [26].

In the literature, cases of thromboembolism in patients with congenital quantitative fibrinogen disorders who had been administered fibrinogen concentrate have been described. We must emphasize that there is no clear evidence of a direct relationship between administration of fibrinogen concentrate and the development of thrombosis. It has been reported that, after administration of fibrinogen concentrate, there is a “collision” with circulating thrombin, which leads to vascular occlusive and minor embolus [49]. On the contrary, there are reports of a number of patients who had been administered fibrinogen long-term without experiencing any thrombosis [50].

Typical epidemiological risk factors for thrombosis include smoking, hypertension, obesity, and the use of oral contraceptives [51]. Thrombophilic mutations (i.e., factor V Leiden and prothrombin G20210A mutations) have also been reported in a small number of patients with congenital quantitative fibrinogen disorders. Thrombosis in more than 30% of patients have been described after surgery, trauma, postpartum, and puerperium. Studies suggest that these risk factors are comparable to those present in the population without CFD [44]. 

To conclude, we have to mention the seminal paper by Peyvandi et al. who stated that, in fibrinogen deficiency, there is a strong relationship between the fibrinogen converting activity to the fibrin level and the clinical bleeding phenotype, even though correlations between genotype and phenotype are difficult to establish. Furthermore, some mutations may increase the bleeding tendency while others may predispose to thrombosis [52]. In the literature, there have been five causal genetic variants reported in dysfibrinogenemia related to thrombophilia (Fibrinogen Caracas V, Vlissingen, Melun, Naples, and Dusart), associated with various pathogenic mechanisms including structural changes in the fibrin network, higher thrombin levels due to impaired fibrinogen binding, decreased fibrinolysis resulting from impaired binding of tissue-type plasminogen activator, or plasminogen to dysfunctional fibrinogen [53]. In particular, fibrinogen Dusart (Aα-Arg554Cys) is one of the mutations where the thrombotic phenotype is better known. The literature describes severe thrombotic events, high incidence of thrombotic embolism, and abnormal fibrin polymerization. This mutation increases the brittleness of blood clots so that they break easily and cause embolism [54]. Another of the causal thrombogenic mutation is Fibrinogen Naples I (Bβ-Ala68Thr), which is characterized by defective thrombin binding and fibrinopeptide cleavage at the fibrinogen substrate site. Functional studies have demostrated that the fibrin networks showed relatively wide fiber bundles likely due to slowed fibrin assembly secondary to delayed fibrinopeptide release. Exclusively homozygous carriers showed impaired binding with high affinity for thrombin [55].

## 4. Laboratory and Genetic Analysis of Congenital Quantitative Fibrinogen Disorders

### 4.1. Laboratory Analyses

Initial screening tests for afibrinogenemia and hypofibrinogenemia should include fibrinogen plasma concentration, measured functionally and immunochemically, prothrombin time (PT), activated partial thromboplastin time (APTT), and thrombin time (TT) [56]. In addition, we have the opportunity to investigate the prothrombin time-derived fibrinogen (PT-Fbg). This time represents an indirect measurement of fibrinogen derived from the change in light transmission or scatter from a PT curve [27]. Genetic analysis should then be performed in order to confirm the diagnosis while screening first-degree relatives in the family. Subsequently, the genotype should be compared with the clinical phenotype, particularly in the case of thrombotic dysfibrinogenemic variants [57].

The diagnosis of afibrinogenemia is established on the undetectable level of functioning fibrinogen and absence or trace amounts of immunoreactive fibrinogen [58]. 

All coagulation tests depending on the formation of fibrin as the last step in the coagulation pathway, to be more exact, PT, APTT, and TT are infinitely prolonged. Plasma activity of all other coagulation factors is physiological [59]. Some abnormalities in platelet-function tests can be observed including abnormalities that are almost completely reversed after the substitution of fibrinogen (platelet adhesion and adenosine diphosphate (ADP)-induced platelet aggregation). On the other hand, thrombin-stimulated and collagen-stimulated platelet aggregation is normal [60]. 

In afibrinogenemia and hypofibrinogenemia, there is a concomitant decrease in fibrinogen antigen tested by immunoassay, gravimetric assays, or by measurement of dry clot weight [61]. Rotational thromboelastography was also proposed as a universal method for monitoring the reaction to administered fibrinogen in subjects with fibrinogen deficiency [33,62]. Maximum clot firmness (one of its parameters) can confirm the effectiveness and safety of normalization of clot formation after the infusion of fibrinogen [33,63,64]. 

### 4.2. Genetic Analyses

The pathogenesis of afibrinogenemia at molecular level has long been clarified. It represents an autosomal recessive disorder [32] with heterozygote patients being without any clinical manifestation and identifiable as hypofibrinogenemic [1]. Afibrinogenemia is the consequence of bialleic mutations in the homozygous or compound heterozygous state in one of genes encoding for the fibrinogen chains. These mutations can affect the synthesis, assembly, intracellular processing, stability, or secretion of the hexameric molecule [65,66]. The spectrum of mutations involved in quantitative fibrinogen disorders (afibrinogenemia and hypofibrinogenemia) involves large deletions, point mutations leading to the occurence of premature termination codons, and missense mutations influencing fibrinogen assembly and/or secretion [43]. The Human Gene Mutation Database (HGMD) (http://www.hgmd.cf.ac.uk/ac/index.php) and database of the Groupe d’Etude sur l’Hémostase de la Thrombose (GEHT) (www.geht.org/databaseang/fibrinogen/) include the spectrum of mutations localized on the *FGA/FGB/FGG* genes (missense/nonsense, splicing, regulatory mutations, small deletions/insertions/indels, and gross deletions/insertions/duplications) [1].

## 5. Case Reports of Mutations Located in the Beta and Gamma Nodules of Fibrinogen Bβ and γ Chains Associated with Thrombotic Complications

We searched for human fibrinogen variants in the GEHT database. All new variants in all three genes *FGA*, *FGB*, and *FGG* enconding of congenital fibrinogen disorders are added regularly to this database. Currently, the GEHT database reports 1,215 molecular abnormalities of fibrinogen (626 in the *FGA* gene, 154 in the *FGB* gene, and 435 in the *FGG* gene).

**Figure 2:**
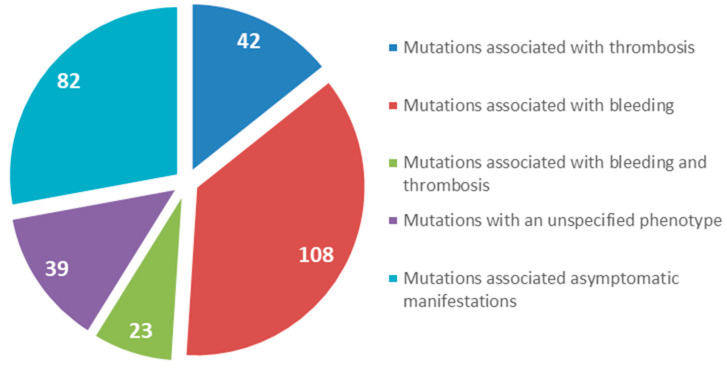
Total number of mutations located in the beta and gamma nodules in the fibrinogen molecule (Bβ and γ chains).

**Figure 3:**
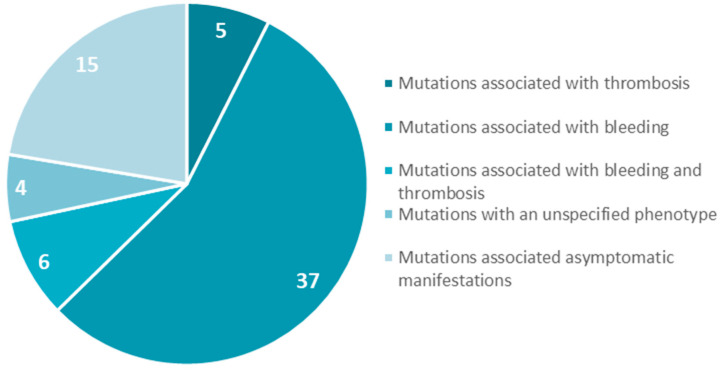
Mutations in exons 5–8 of the *FGB* gene encoded the fibrinogen Bβ chain.

**Figure 4:**
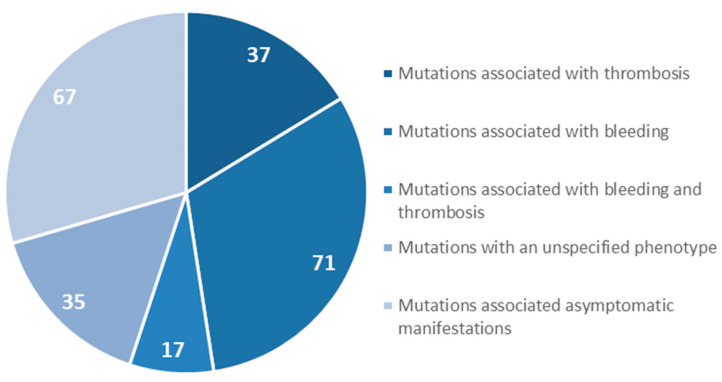
Mutations in exons 5–9 of the *FGG* gene encoded the fibrinogen γ chain.

We focused on gene mutations in the beta and gamma nodules of the Bβ and γ chains, which are responsible for congenital quantitative fibrinogen disorders associated with thrombosis, considering that, in the last two years, we have identified two novel mutations in hypofibrinogenemia [5,26] associated with the thrombotic phenotype, which are both located in the beta and gamma nodules of fibrinogen Bβ and γ chains. The beta and gamma nodules in the fibrinogen molecule are encoded by exons 5–8 of the *FGB* gene and by exons 5–9 of the *FGG* gene. Overall, in the GEHT database, we found 294 mutations in these exons (Figure 2). In Figure 3 and Figure 4, all genetic variants in the exons of the *FGB* and *FGG* genes encoding the beta and gamma nodules are summarized.

Among the 294 mutations identified in the exons coding for the beta and gamma nodules in the fibrinogen molecule, 42 were associated exclusively with thrombotic complications in quantitative and qualitative fibrinogen disorders. Among these 42 mutations, 80% were located in the *FGG* gene, but only 11 were identified in patients with congenital afibrinogenemia and hypofibrinogenemia. Seven mutations were located in the *FGB* gene and four mutations were located in the *FGG* gene. Though these figures should be considered with caution, the fact that we observe a higher number of mutations in the beta nodule than in the gamma nodule is puzzling. The main driver of fibrin polymerization is a strong knob ‘A’ binding to hole ‘a’ in the gamma nodule, whereas the B:b interactions appear to be less important, so that we would expect a higher incidence of mutations in the gamma rather than in the beta nodule. 

**Figure 5:**
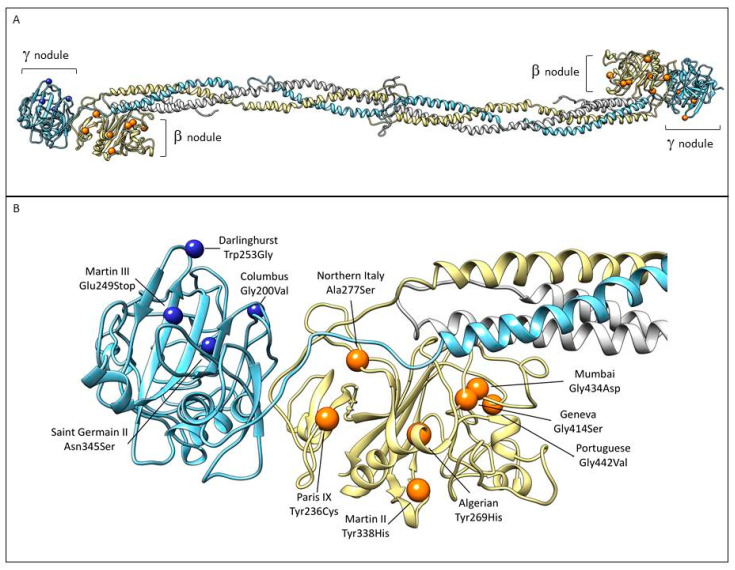
Localization of the mutations associated with thrombosis within the beta and gamma nodules in the fibrinogen structure. (**A**) The three chains are colored as in Figure 1. Mutations associated with thrombotic complications in β nodule (orange sphere) and γ nodule (blue spheres) are indicated (Table 2). (**B**) Close-up view of the β and γ nodules and the described mutations. Images were produced using UCSF Chimera package (http://www.rbvi.ucsf.edu/chimera) [73] and the 3GHG coordinates.

In this case, we will focus on all case reports and clinical studies describing patients with afibrinogenemia and hypofibrinogenemia. We will subdivide the case studies into two parts: mutations in the *FGB* and *FGG* gene (all listed in Table 2). Figure 5 lists the localization of the mutations associated with thrombosis within the beta and gamma nodules in molecule fibrinogen.

## 6. Mutations in the *FGB* Gene

### 6.1. Fibrinogen PARIS IX

A 36-year-old women with moderate hypofibrinogenemia (Fbg function: 0.7 g/L) with recurrent epistaxis during childhood. In her personal history, easy bruising and menorrhagia were present. The patient delivered a dead fetus by emergency Caesarean section at 20 weaks of pregnancy due to placental abruption. She developed a distal vein thrombosis 10 days after the Caesarean section. Genetic analysis identified both a heterozygous missense mutation in exon 5 of the *FGB* gene c.5909A>G (p.Tyr266Cys) and a heterozygous IVS7+1G>C transversion, which possibly affects exon 8 splicing. A nonsense mutation (p.Tyr266Stop) was previously described, which affects the same p.Tyr266 residue. Yet, carriers were characterized by normal fibrinogen levels. The p.Tyr266Stop mutation was associated with pulmonary embolism (PE) and not with hemorrhagic manifestations. 

The p.Tyr266Cys mutation occurs in the second β sheet and may destabilize the structure of fibrinogen. There exist the possibility that abnormal disulfide bonding occurs in the mutated chain [74].

### 6.2. Fibrinogen ALGERIAN

A 30-year-old patient with afibrinogenemia from a consanguineous Algerian family. The patient has been identified because of an unprovoked pulmonary embolism (PE) with the need for hospitalization. In childhood, the first signs of the disease were prolonged bleeding during circumcision. During childhood, he overcame severe bleeding events requiring transfusion therapy. Genetic analysis revealed a homozygous missense mutation in the fibrinogen Bβ chain: c.895T>C (exon 6), p.Tyr299His. His brother had similar biologic findings, but DNA and other clinical data were unavailable. The thrombophilia screening revealed the heterozygous Factor V Leiden mutation. The p.Tyr299His missense mutation is predicted to convert a core hydrophobic to a basic amino acid. This change could lead to incorrect misfolding of the βC domain and to the absence of the hexameric fibrinogen molecule [48].

### 6.3. Fibrinogen NORTHERN ITALY

A 41-year-old patient from Northern Italy without family or personal history of bleeding was diagnosed with afibrinogenemia occasionally when he was 20 years old. At the age of 36 years, he was diagnosed with myocardial infarction (MI) and, six days later, he suffered from ischemic stroke and arterial thrombosis (right radial artery). At that time, fibrinogen levels were undetectable. All thrombophilia tests were negative. Genetic analysis discovered a novel homozygous missense mutation c.919G>T in exon 6 of the *FGB* gene (p.Ala307Ser). This mutation was confirmed in the mother in the heterozygous state. The mutation converts a nonpolar residue into a slightly polar one and modify the fibrinogen structure. The replacement of the amino-acid alanine with serine can lead to creation of an extra N-glycosylation site at the 305 asparaginase residue. This may allow for an incorrect folding and assembling of mature multimeric protein, and determines an intracelular degradation [67].

### 6.4. Fibrinogen MARTIN II

A 62-year-old man with severe hypofibrinogenemia. His personal history was characterized by non-provoked recurrent deep and superficial venous thrombosis of the right and left leg. This patient did not have significant bleeding episodes in his history throughout a lifetime, including perioperative bleeding and anticoagulant treatment. The patient’s 34-year-old son also overcame spontaneous recurrent deep-vein thrombosis (DVT) of the lower limbs. The results of the testing for a thrombophilic state were negative in the patient and his son. In case of the index patient, a novel homozygous missense mutation located in exon 7 of the fibrinogen Bβ-chain gene at nucleotide position c.1102T>C (p.Tyr368His) was found. The genetic screening detected this mutation in the heterozygous state in all his three children (one daughter, two sons) who were diagnosed with a mild hypofibrinogenemia. The switch of an uncharged aromatic amino-acid side chain with a positively charged residue is likely to interfere with the correct binding of the βC domain by modifying the delicate balance in the distribution of hydrophobic and hydrophilic regions. This change can result in the incorrect composition of the βC domain and reduction of fibrinogen hexamer secretion compatible with the observed hypofibrinogenemia [26,33].

### 6.5. Fibrinogen GENEVA

A young boy with afibrinogenemia was identified as a compound heterozygote for two mutations in the *FGB* gene. His first clinical manifestations were two days after birth with the development of bilateral cephalohematomas. Bleeding was treated with cryoprecipitate infusions and regular infusion of a fibrinogen concentrate across the central line. However, management of treatment was complicated by a complete and asymptomatic thrombosis of the upper venous system. From his family history, researchers registered that his father died at 34 years of age from a presumed MI. Sequence analysis of the *FGB* gene revealed the presence of both a missense mutation in exon 8: c.1330G>C (p.Gly444Ser) and a nonsense mutation in exon 2: c.139C>T (p.Arg47Stop). The father was a heterozygous carrier of the p.Gly444Ser and the mother was a heterozygous carrier of the p.Arg47Stop mutation. Both parents (father: 1.48 mg/mL, mother: 2.0 mg/mL) had normal or intermediate fibrinogen levels. The molecular mechanism underlying the fibrinoogen deficiency was investigated by co-expressing the mutant *FGB* cDNA together with the wild-type *FGA* and *FGG* cDNAs. These experiments demonstrated that the fibrinogen molecules containing the mutant β chain were able to assemble but were not secreted into the media [68].

### 6.6. Fibrinogen MUMBAI

A 28-year-old woman with afibrinogenemia was studied. Doppler studies revealed a complete thrombosis of the portal and splenic veins, which were replaced by multiple collaterals forming a portal cavernoma. The right hepatic vein was severely thrombosed. In the liver, any significant finding was reported. During a lifetime, several hemorrhagic manifestations also appeared including umbilical cord bleeding at birth, ecchymosis, hematemesis, menorrhagia, and prolonged bleeding from cuts (leading, in some cases, to anemia). The genetic analysis confirmed the presence of a homozygous missense mutation c.1391G>A (p.Gly464Asp) in exon 8 of the *FGB* gene. In vitro expression, experiments demonstrated normal synthesis but intracellular retention of the mutant fibrinogen and reduction of fibrinogen secretion [69].

### 6.7. Fibrinogen PORTUGUESE

A 49-year-old Portuguese women with a severe hypofibrinogenemia reported two pregnancy-related thrombosis, i.e., deep vein thrombosis (DVT) of the left leg, and PE and one unprovoked DVT of the right leg. She had two miscarriages and two pregnancies without complications. Screening for thrombophilic mutations was negative. No bleeding manifestations were reported. DNA analysis identified in exon 8 of the *FGB* gene the homozygous missense mutation c.1415G>T (p.Gly472Val). Protein modeling did not observe any changes in the 10-Å region surrounding the mutation site. This modest effect on the structure of the βC domain may explain that this homozygous mutation leads to hypofibrinogenemia rather than afibrinogenemia with a probable partial secretion of the mutant molecule [48].

## 7. Mutations in the *FGG* Gene

### 7.1. Fibrinogen COLUMBUS

A 2-year-old boy was diagnosed with severe hypofibrinogenemia after birth. His twin had diagnosed subdural and subarachnoid hemorrhaging and diffuse areas of hypoxic ischemia postpartum. The twin died at 7 months of age due to an intracranial thrombotic episode (intracranial sinus thrombosis, diffuse hypoxic ischemia) associated with hemorrhagic events. The thrombophilic screen confirmed, for the boy and his mother, the presence of the factor V Leiden and MTHFR C677T variants (both at the heterozygous state). At the same time, the genetic analysis of all fibrinogen genes confirmed the presence of a heterozygous missense mutation in exon 7 of the *FGG* gene, i.e., c.677G>T (p.Gly226Val). Protein modeling showed that the novel missense mutation lies after the first strand of the five-stranded β sheet of the gamma nodules, and that the p.Gly226 residue is in close connection with the p.Tyr374 amino acid. Since the p.Gly226 residue is solvent exposed and allows the p.Tyr374 side chain to well accommodate in the region, the mutation, by introducing the hydrophobic amino-acid valine, which is likely to destabilise the tertiary structure of the molecule. Therefore, this mutation may contribute to the cumulative effect of other frequent thrombophilic mutations [70].

### 7.2. Fibrinogen MARTIN III

A 45-year-old male was diagnosed with a mild hypofibrinogenemia upon investigation for recurrent non-provoked DVT of the leg. The patient did not report any significant bleeding episode during a lifetime. The screening tests for thrombophilia (factor V Leiden and prothrombin G20210A mutations) were negative. However, scientists observed higher levels the of coagulation factor VIII (204%, normal range: 60–150%). Genetic analysis revealed a heterozygous nonsense mutation in exon 7 of the *FGG* gene, c.823G>T (p.Glu275Stop). The C-terminal domain of the γ chain is critical for fibrinogen secretion from hepatocytes [75]. With these premises, it is highly probable that fibrinogen molecules bearing this nonsense mutation could not be competent for secretion. At the same time, alternative mechanisms, by which some transcripts skip the stop codon (exon skipping, alternative splicing, incorrect codon encoding) in such a way that they have secreted fibrinogen with thrombotic potential, are not excluded [5].

### 7.3. Fibrinogen DARLINGHURST

The proband was a Turkish female with severe hypofibrinogenemia with a history of multiple miscarriages, bleeding, and thrombosis. She was examined for symptoms of right heart failure and secondary plumonary hypertension due to chronic thromboembolic disease. DNA sequencing identified a homozygous missense mutation in exon 7 of the *FGG* gene c.835T>G (p.Trp279Gly). Her son was diagnosed with a mild hypofibrinogenemia with an unspecified clinical phenotype. He carried the same missense mutation in the heterozygous state. The mutation could be responsible for protein resistance to lysis, which possibly explains the thrombosis in the patient, especially as no other thrombophillic risk factors were found [71].

### 7.4. Fibrinogen SAINT–GERMAIN II

A 26-year-old man with a moderate hypofibrinogenemia experienced extensive DVT of the left leg associated with PE. The screening tests for thrombophilic mutations showed a heterozygous factor V Leiden mutation and heterozygous prothrombin G20210A mutation. Patient´s mother was also diagnosed as moderate hypofibrinogenemic and she became a carrier in the heterozygous state of the prothrombin G20210A variant. Sequencing of fibrinogen genes in the patient and his mother identified the heterozygous missense mutation c.7687A>G in exon 8 of the *FGG* gene (p.Asn371Ser). Results of this study suggested that fibrinogen molecules containing an abnormal γ chain are absent in plasma, or, at most, are present in trace amounts. p.Asn371 is located near the center of the Dγ subdomain. This residue may be important for forming hydrogen bonds with p.230Phe and p.237Tyr that stabilize the folding in this region. In its absence, the region may be rendered incapable of a proper assembly or secretion from the liver. However, the authors of the study did not find evidence to suggest hepatic retention of fibrinogen in their patients [72].

## 8. Conclusions

Fibrinogen plays a key role in the process of hemostasis. Congenital quantitative fibrinogen disorders (afibrinogenemia, hypofibrinogenemia) are rare bleeding disorders, but thrombotic complications can be present [44,76]. Although thrombosis in these patients is uncommon, in the literature, several cases were reported. There are known risk factors for thrombosis such as surgery, trauma, pregnancy, postpartum period, replacement of fibrinogen concentrate, and causal thrombophilic mutations [77]. However, in most cases, the thrombosis is spontaneous [26]. The true mechanisms in patients with quantitative fibrinogen disorders remain to be elucidated. Due to the low prevalence of hypofibrinogenemia and afibrinogenemia, there is little information on pathophysiology or optimal treatment of thrombosis in these patients. Managing patients with CFD with thrombotic events is challenging as anticoagulant treatment may exacerbate the underlying bleeding risk, which can be life-threatening [33]. We hope that this article will generate additional interest in investigators who may eventually contribute to the better understanding of the development of thromboembolic complications in patients with a low level of fibrinogen. Great emphasis on thrombosescharacterization of novel molecular defects responsible for fibrinogen deficiency combined with different types of phenotypes will continue to provide a better comprehension of the complexity of genetic background that predisposes patients to fibrinogen disorders and thromboembolic complications, and can help us toward the early identification of patients at risk of thrombosis and allow us to better manage the disease and minimize the risk associated with it.

## Figures and Tables

**Table 1:** Classification of congenital fibrinogen disorders [27].

Type and Subtypes	Descriptions
**Afibrinogenemia**	
Afibrinogenemia	Afibrinogenemia and bleeding phenotype or asymptomatic individuals
**A.** Afibrinogenemia with a thrombotic phenotype	Afibrinogenemia and thrombotic phenotype
**Hypofibrinogenemia**
**A.** Severe hypofibrinogenemia	Functional fibrinogen level ˂ 0.5 g/L
**B.** Moderate hypofibrinogenemia	Functional fibrinogen level between 0.5–0.9 g/L
**C.** Mild hypofibrinogenemia	Functional fibrinogen level between 1 g/L and lower limit of normal value
**D.** Hypofibrinogenemia with fibrinogen storage disease	Familial hypofibrinogenemia with histologically proven accumulation of fibrin in hepatocytes
**Dysfibrinogenemia**	
**A.** Dysfibrinogenemia	Dysfibrinogenemia and bleeding phenotype, thrombotic phenotype or asymptomatic individuals
**B.** Thrombotic-related dysfibrinogenemia	Dysfibrinogenemia patients carriers of a thrombotic fibrinogen mutation * or suffering from thrombotic events with a first-degree familial thrombotic history (relatives with the same genotype) without any other thrombophilia
**Hypodysfibrinogenema**	
**A.** Severehypodysfibrinogenemia	Antigenic fibrinogen level ˂ 0.5 g/L
**B.** Moderate hypodysfibrinogenemia	Antigenic fibrinogen level between 0.5–0.9 g/L
**C.** Mild hypodysfibrinogenemia	Antigenic fibrinogen level between 1 g/L and lower limit of normal value

* Fibrinogen Dusart, Fibrinogen Caracas V, Fibrinogen Ijmuiden, Fibrinogen New York I, Fibrinogen Nijmegen, Fibrinogen Naples at homozygous state, Fibrinogen Melun.

**Table 2:** Description of clinical studies and mutations in exons of *FGB* and *FGG* genes encoding beta and gamma nodules of fibrinogen molecule associated with thrombotic complications.

Name/Origin	Plasma Protein	Native Protein	Gene	Gene Status	Type	Haemorraghes	Numbers of Studied Family Member/Positive Numbers of Mutation	Numbers of Thrombotic Complications	Other Thrombophilic States	References
**Fibrinogen Bβ Chain Mutations Associated with Thrombosis**										
**PARIS IX**	Bβ(236) Tyr>Cys	p.Tyr266Cys	5909A>G IVS7+1G>C	Compound	Hypofib.	Yes	2/1	2	Not listed	[6]
**ALGERIAN**	Bβ(269) Tyr>His	p.Tyr299His	c.895T>C	Homozyg.	Afib.	Yes	1/1	1	Heterozygous Factor V Leiden mutation	[48]
**NORTHERN ITALY**	Bβ(277) Ala>Ser	p.Ala307Ser	c.919G>T	Homozyg.	Afib.	No	3/1	3	No other thrombophilic state	[67]
**MARTIN II**	Bβ(338) Tyr>His	p.Tyr368His	c.1102T>C	Homozyg.	Hypofib.	No	4/2	5	No other thrombophilic state	[26]
**GENEVA**	Bβ(17) Arg>Stop Bβ(414) Gly>Ser	p.Arg47Stop p.Gly444Ser	c.139C>T c.1330G>C	Compound	Afib.	Yes	4/2	2	No other thrombophilic state	[68]
**MUMBAI**	Bβ(434) Gly>Asp	p.Gly464Asp	c.G1391A	Homozyg.	Afib.	Yes	1/1	1	Heterozygous PAI 4G/5G polymorphism	[69]
**PORTUGUESE**	Bβ(442) Gly>Val	p.Gly472Val	c.1415G>T	Homozyg.	Hypofib.	No	1/1	5	No other thrombophilic state	[48]
**Fibrinogen γ Chain Mutations Associated with Thrombosis**										
**COLUMBUS**	γ(200) Gly>Val	p.Gly226Val	c.677G>T	Heterozyg.	Hypofib.	Yes	8/2	2	Heterozygous Factor V Leiden, MTHFR C677T mutations	[70]
**MARTIN III**	γ(249) Glu>Stop	p.Glu275Stop	c.823G>T	Heterozyg.	Hypofib.	No	1/1	3	No other thrombophilic state	[5]
**DARLINGHURST**	γ(253) Trp>Gly	p.Trp279Gly	c.835T>G	Homozyg.	Hypofib.	Yes	2/1	2	No other thrombophilic state	[71]
**SAINT GERMAIN II**	γ(345) Asn>Ser	p.Asn371Ser	7687A>G	Heterozyg.	Hypofib.	No	3/1	2	Heterozygous Factor V Leiden mutation, prothrombin G20210 mutation	[72]

Afib, afibrinogenemia; Heterozyg, heterozygous; Heterozyg, heterozygous; Hypofibt, hypofibrinogenemia; PAI, plasminogen activator inhibitor-1.
